# Radiation-induced Akt activation modulates radioresistance in human glioblastoma cells

**DOI:** 10.1186/1748-717X-4-43

**Published:** 2009-10-14

**Authors:** Hui-Fang Li, Jung-Sik Kim, Todd Waldman

**Affiliations:** 1 Department of Oncology, Lombardi Comprehensive Cancer Center, Georgetown University School of Medicine, Washington, DC, USA

## Abstract

**Background:**

Ionizing radiation (IR) therapy is a primary treatment for glioblastoma multiforme (GBM), a common and devastating brain tumor in humans. IR has been shown to induce PI3K-Akt activation in many cell types, and activation of the PI3K-Akt signaling pathway has been correlated with radioresistance.

**Methods:**

Initially, the effects of IR on Akt activation were assessed in multiple human GBM cell lines. Next, to evaluate a potential causative role of IR-induced Akt activation on radiosensitivity, Akt activation was inhibited during IR with several complementary genetic and pharmacological approaches, and radiosensitivity measured using clonogenic survival assays.

**Results:**

Three of the eight cell lines tested demonstrated IR-induced Akt activation. Further studies revealed that IR-induced Akt activation was dependent upon the presence of a serum factor, and could be inhibited by the EGFR inhibitor AG1478. Inhibition of PI3K activation with LY294002, or with inducible wild-type PTEN, inhibition of EGFR, as well as direct inhibition of Akt with two Akt inhibitors during irradiation increased the radiosensitivity of U87MG cells.

**Conclusion:**

These results suggest that Akt may be a central player in a feedback loop whereby activation of Akt induced by IR increases radioresistance of GBM cells. Targeting the Akt signaling pathway may have important therapeutic implications when used in combination with IR in the treatment of a subset of brain tumor patients.

## Background

Glioblastoma multiforme (GBM), or grade IV astrocytoma, is the most common and lethal primary malignant brain tumor in humans [1-3]. Despite surgical resection and treatment with ionizing radiation (IR) and temozolamide, the median survival for GBM patients is approximately 1 year [2,3]. Virtually all patients suffer tumor recurrence despite aggressive irradiation, emphasizing the radioresistant nature of GBMs. As such, understanding the molecular mechanism of radioresistance is essential for developing more effective radiotherapy treatment regimens for GBM.

The PI3K-Akt signaling pathway is a ubiquitous and evolutionarily conserved signaling cascade that is involved in numerous cellular functions, including apoptosis, cell proliferation, differentiation, migration, and metabolism [4,5]. Activation of PI3K-Akt signaling is associated with poor prognosis in multiple tumor types, including GBMs [6,7]. PI3K is coupled with a variety of growth factor-dependent receptor tyrosine kinases, such as epidermal growth factor receptor (EGFR), insulin-like growth factor receptor, platelet-derived growth factor receptor, and insulin receptor [8-10]. Upon stimulation of its upstream receptors, PI3K is activated and generates phosphatidylinositol (3,4,5) P_2 _(PIP_3_). PIP_3 _is converted to inactive phosphatidylinositol (4,5) P_2 _(PIP_2_) by the PTEN lipid phosphatase, which is commonly deleted or mutated in GBM [7,11,12].

The most important downstream effector of PI3K signaling is the serine/threonine kinase Akt (also known as PKB). There are three closely related Akt isoforms in mammalian cells, including Akt1 (PKBα), Akt2 (PKBβ), Akt3 (PKBγ) [4]. All Akt isoforms bind to PIP_3 _through pleckstrin-homology (PH) domains, and translocate to the plasma membrane where they are activated via phosphorylation at residues Ser473 and Thr308. Once activated, Akt promotes cellular proliferation and inhibits apoptosis through phosphorylation of multiple substrates, including caspase-9, Bad, GSK3, and forkhead transcription factors, such as FKHR (FOX1), FKHRL (FOXO3), and AFX (FOXO4) [5,13].

Activation of PI3K-Akt signaling is important in most human malignancies, including hematopoietic, melanoma, non-small cell lung, pancreatic, endometrial and ovarian, breast, prostate, hepatocellular, and brain cancers [4,7,11]. PTEN, the primary negative regulator of the PI3K-Akt signaling pathway, is an important tumor suppressor. Deletions or inactivating mutations of PTEN are found in various cancer specimens, cancer cell lines, and inherited cancer predisposition syndromes, making PTEN one of the most commonly inactivated tumor suppressor genes in human cancer [12,14]. Recently, mutations in PIK3CA (encoding the catalytic subunit of PI3K, P110α) were observed in multiple cancers, including brain tumors, further supporting the fundamental role of PI3K pathway activation in the pathogenesis of human cancer [15,16].

PTEN is among the most frequently mutated or deleted tumor suppressor genes in GBM, as genetic and epigenetic alterations have been identified in at least 60% of patients [7]. Importantly, the role of PI3K-Akt signaling in gliomagenesis has been demonstrated in both animal and cell culture models. Activating Akt by deletion of PTEN or by Myr-Akt (constitutively active Akt) expression has been shown to increase tumor incidence, accelerate tumor onset, and elevate tumor malignancy in multiple mouse glioma models [17,18]. Akt activation is also crucial for the transformation of human astrocytes *in vitro *[7,19], and EGFR, an upstream regulator of PI3K-Akt signaling, is also commonly activated in GBM [7,16,20].

Activation of the PI3K-Akt signaling pathway is associated with radioresistance in many cancers, including those of the colon, bladder, prostate, head and neck, cervix, and brain [21,22]. Inhibition of the PI3K-Akt pathway has been shown to impair DNA repair after IR [23,24], and result in radiosensitization in a variety of different cell types including human GBMs [22,25] For example, inhibition of PI3K-Akt pathway via treatment with PI3K inhibitors or PTEN expression has been shown to increase radiosensitivity in human GBM cells [26,27]. Although most reports indicate that inhibition of Akt activation reduces radiosensitivity, a report from del la Pena et al showed little or no effect of Akt activation on the effectiveness of IR treatment in a number of human GBM cell lines [28].

Importantly, IR has been shown to induce Akt activation in multiple cell types, including some human GBM cells [29-31]. In this study, we investigated PI3K-Akt activation following irradiation in multiple GBM cell lines, and assessed its effect on the ability of human gliobastoma cell lines to respond to IR treatment. To evaluate the effect of IR induced Akt activation on radiosensitivity, Akt activation was inhibited during IR with various genetic and pharmacological approaches. We found that pharmacologic and genetic inhibition of PI3K activity, as well as direct pharmacological inhibition of EGFR and Akt led to increased radiosensitivity of human GBM cells.

## Methods

### Cell culture and reagents

U87MG, MO59J, LN18, H4, A172, DBTRG-05MG, LN229, and HS683 cells were obtained from the American Type Culture Collection, and were cultured in Dulbecco's modified Eagle's medium (Invitrogen) supplemented with 10% FBS and 1% penicillin/streptomycin. U87MG cells containing transgenes for inducible wild-type PTEN, or the phosphatase-inactive mutant form of PTEN, PTEN-C124S, were gifts from Dr. Georgescu [32], and were grown in Dulbecco's modified Eagle's medium containing 0.5 mg/mL G418, 10 μg/mL blasticidin (Invitrogen), 10% FBS, and 1% penicillin/streptomycin. All cells were incubated at 37°C in 5% CO_2_. LY294002 and doxycycline were purchased from Sigma, AG1478 from Biosource, SH-5 from Calbiochem, and MK-2206 from Selleck Chemicals.

### Irradiation

Sub-confluent cell monolayers were irradiated using a J.L. Shepard Mark I ^137^Cs irradiator at ~2 Gy/min.

### Western blot analysis

Cells were lysed in lysis buffer (Cell Signaling) containing 20 mM Tris-HCl (pH 7.5), 150 mM NaCl, 1 mM EDTA, 1 mM EGTA, 1% Triton X-100, 2.5 mM sodium pyrophosphate, 1 mM β-glycerophosphate, 1 mM Na_3_VO_4_, 1 μg/ml leupeptin supplemented with proteinase inhibitor cocktails (Roche) and phosphatase inhibitor cocktails (Sigma). Cell lysates were separated by SDS-PAGE and transferred to PVDF membranes. After probing with primary antibodies, the membranes were incubated with horseradish peroxidase-conjugated secondary antibody, and visualized by ECL (Pierce). Antibodies specific for total Akt and phospho-Akt (Ser473) were obtained from Cell Signaling Technologies. Antibodies specific for PTEN (clone 6H.1) was from Cascade Bioscience, and that for α-tubulin was from Neomarkers.

### Clonogenic Survival Assay

Cells in exponential growth phase were irradiated as described above. Prior to irradiation, cells were treated with LY294002, AG1478, SH-5, or doxycycline as described in the Figure legends. At 4 - 24 hr post-radiation, the cells were detached from the culture dish with trypsin, and were seeded at various dilutions into 25 cm^2 ^tissue culture flasks in normal medium. Colonies were allowed to grow for 14 days before staining with a 0.2% crystal violet/formalin solution, and counted under stereomicroscopy. Colonies were defined as clusters of >50 cells. Colony-forming efficiency is reported as the survival fraction, which is defined as the total number of clones in irradiated cells divided by total number of clones in otherwise identical unirradiated cells. Each point on the survival curve represents the mean surviving fraction from at least three replicates. Cell survival measurements were fitted to a linear quadratic mathematical model using the GraphPad Prism 4 program [33].

## Results

### IR induces Akt phosphorylation in a subset of human GBM celllines

**Figure 1:**
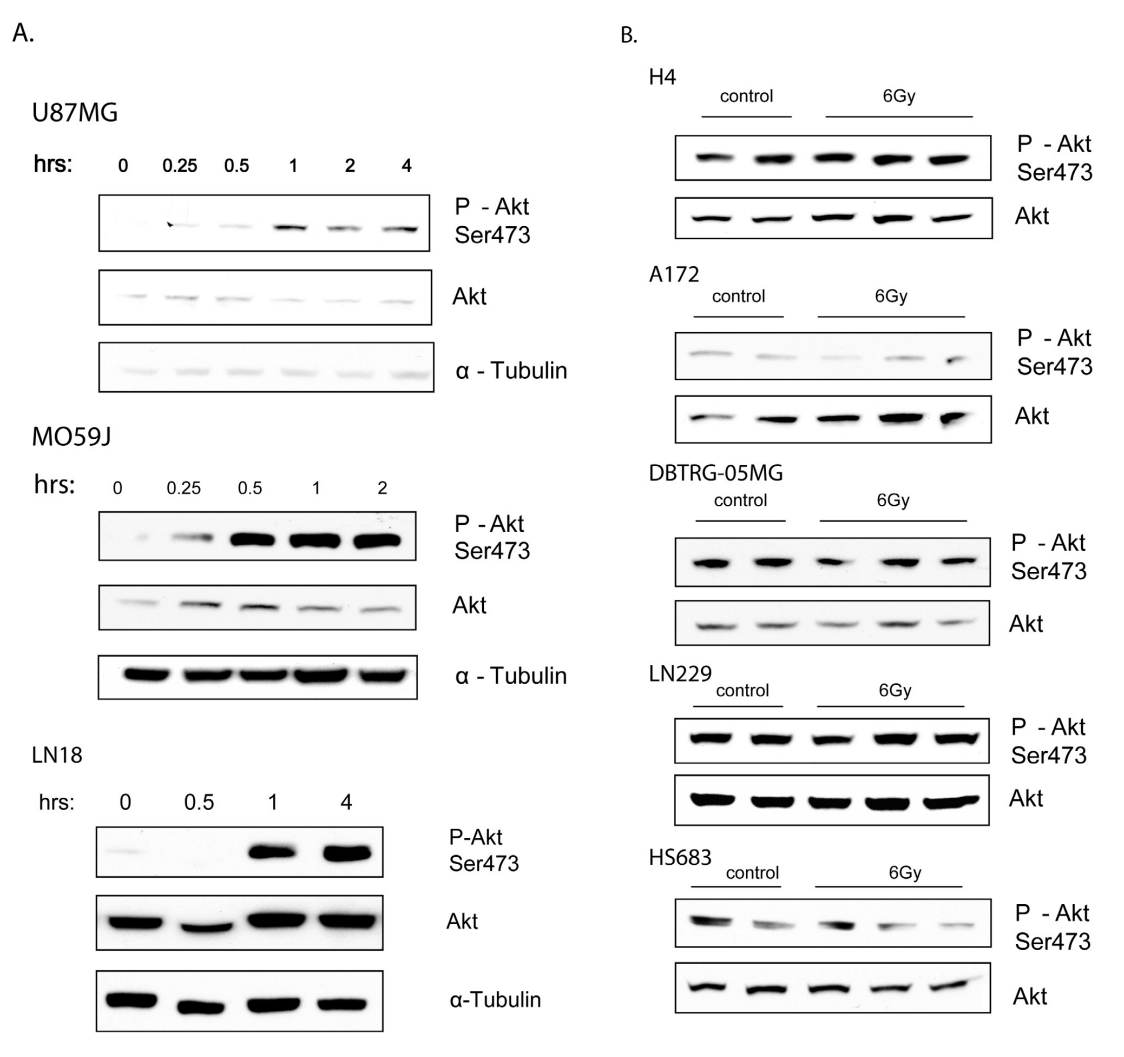
**The effect of IR on Akt phosphorylation differs in human GBM cell lines**. **A**. U87MG, MO59J and LN18 cells were irradiated with 6 Gy and harvested after the indicated times. Cell lysates were prepared and subjected to Western blot analysis with the indicated antibody. **B**. H4, A174, DBTRG-05MG, LN229, and HS683 cells were irradiated with 6 Gy and harvested after 1 hr. Cell lysates were prepared and subjected to Western blot analysis with the indicated antibody.

We began our studies by testing the effect of IR on Akt phosphorylation in eight GBM cell lines. Akt activation was assessed by comparing the levels of basal Akt phosphorylation to that present 1 hr after a single dose of 6 Gy radiation. IR led to increased phosphorylation of Akt in three of the cell lines (U87MG, MO59J, and LN18), which reached maximal levels within 1 hr of IR treatment, and maintained an elevated level for several hours (Fig. 1A, B). From these data we conclude that radiation induces robust but transient phosphorylation of Akt in a subset of human GBM cell lines.

### IR induces Akt activation in U87MG cells via EGFR in a serum factor-dependent manner

U87MG cells, which harbor a mutationally inactivated PTEN gene by virtue of homozygous splice site mutations [34], were chosen for subsequent mechanistic and phenotypic studies. Initially, we performed a dose response curve to identify the optimal dose of IR for maximal induction of Akt phosphorylation. We found that modifying the dose did not enhance Akt phosphorylation (data not shown).

**Figure 2:**
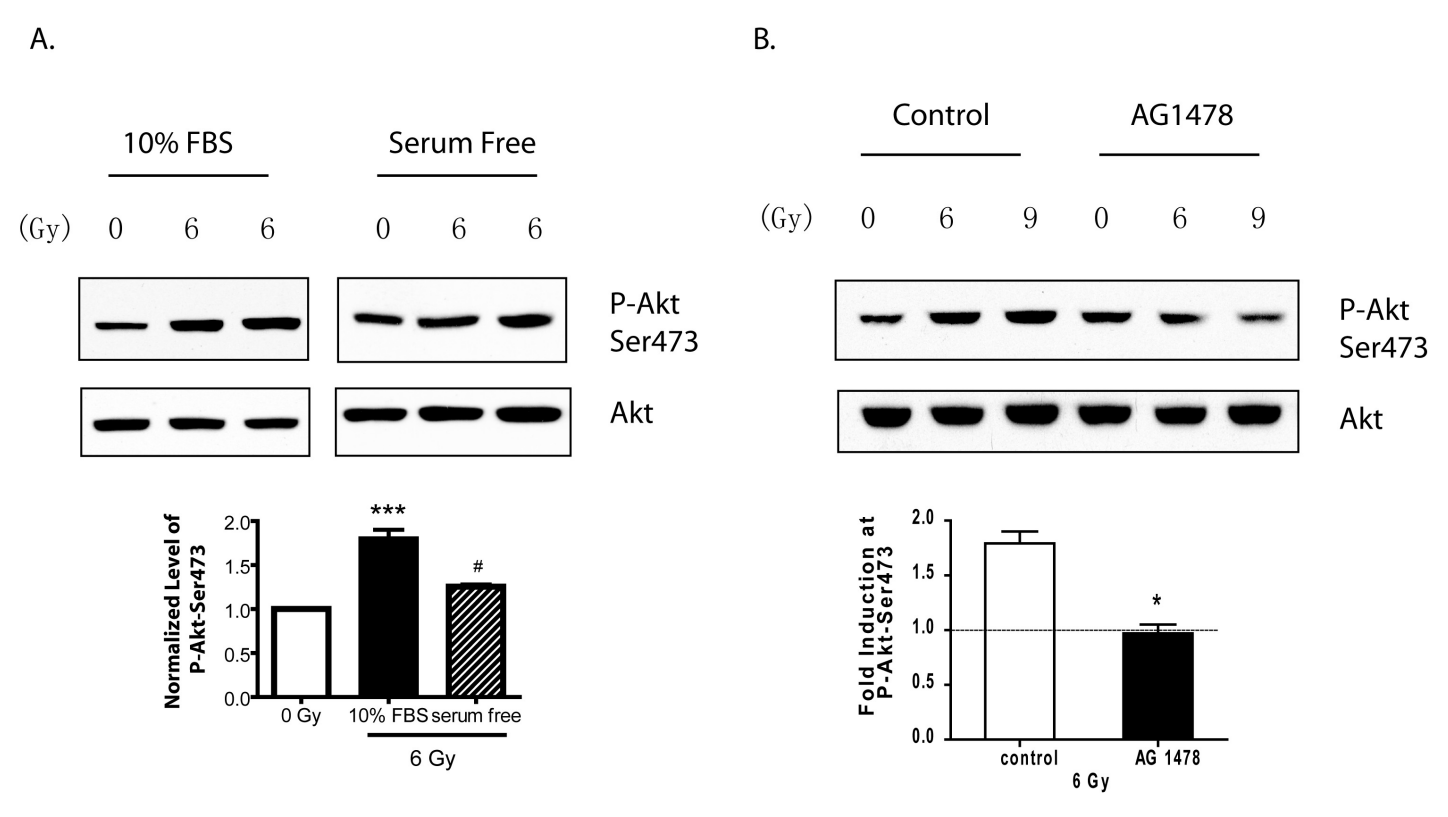
**IR induces Akt activation in U87MG cells through EGFR in a serum factor-dependent manner**. **A**. U87MG cells were cultured in the absence or presence of 10% FBS for 18-20 hr, then irradiated at 6 Gy. Cell lysates were harvested 1 hr later and subjected to Western blot analysis with the indicated antibody. The ratio of P-Akt-Ser473 to total Akt pooled from three different experiments were shown in the lower panel. Results represent mean ± SEM, ***:*p *< 0.001 compared to 0 Gy; #: *p *< 0.05 compared to 10% FBS (one-way ANOVA) **B**. Cells were treated with 5 μM AG1478 for 1 hr, then were irradiated for 1 hr at the indicated dosage. Cell lysates were prepared and subjected to Western blot analysis with the indicated antibody. The fold induction of normalized P-Akt-Ser473 induced by 6 Gy pooled from two different experiments were shown in the lower panel. Results represent mean ± SEM, **:*p *< 0.01(Student's *t*-test).

We next investigated the mechanism of IR-induced Akt phosphorylation, and began by testing for a serum requirement for this effect. As shown in Fig. 2A, cells grown in serum-free conditions displayed attenuated IR-induced Akt phosphorylation, suggesting that a factor present in serum is required for optimal IR-induced Akt phosphorylation.

As EGFR (also known as ErbB1) is commonly activated by genomic amplification in GBM and has previously been implicated in radiation resistance [1,9,10,20,35], we tested if EGFR ligands were the serum factor responsible for IR-induced Akt phosphorylation. Cells were pretreated with the EGFR inhibitor AG1478 for 1 hr, and were then irradiated. Cell lysates were prepared and used in Western Blot analysis for phosphorylated Akt. As shown in Fig. 2B, U87MG cells treated with AG1478 failed to undergo IR-induced Akt activation, indicating that activation of EGFR by IR is required for IR-induced Akt phosphorylation in these cells.

### Pharmacological inhibition of PI3K and EGFR enhances the radiosensitivity of U87MG cells

We next tested if IR-induced Akt signaling modulated the radiosensitivity of GBM cells. First, a PI3K inhibitor was used to inhibit IR induced Akt activation, as PI3K is the upstream signaling molecule for Akt. Cells were pretreated for 1 hr with LY294002, which is a potent inhibitor of PI3K, followed by irradiation at 0 - 9 Gy. The cells were incubated overnight subsequent to removing the drug 4 hr after IR, and their reproductive growth ability was measured using clonogenic survival assay as described in the Methods.

**Figure 3:**
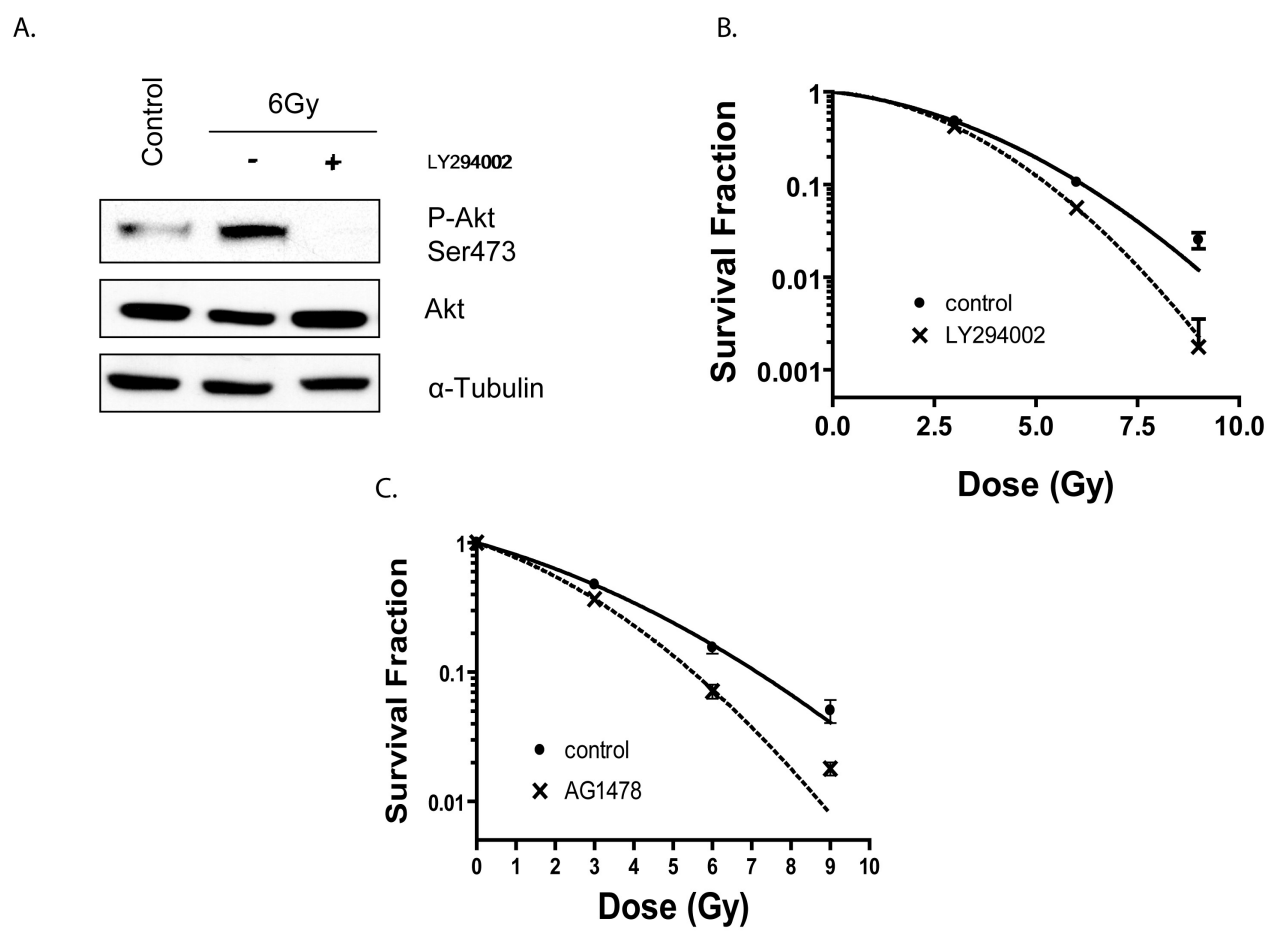
**Inhibition of PI3K-Akt signaling with PI3K inhibitor LY294002 or EGFR inhibitor AG1478 increases the radiosensitivity of U87MG cells**. **A**. U87MG cells were treated with 20 μM LY294002 for 1 hr prior to IR, and then irradiated with 6 Gy. Total cell lysate was harvested 1 hr after IR and subjected to Western blot analysis with the indicated antibody. Cells without IR treatment were used as a control. **B**. Cells were treated with vehicle (control) or 20 μM LY294002 for 1 hr, then irradiated with indicated dosage. 4 hr after IR, cells were fed with drug-free medium, and incubated for another 20 hr at 37°C, after which they were trypsinized and seeded for clonogenic survival assays. Colony-forming efficiency was determined 14 d later. Results were pooled from three different experiments. **C**. Cells were treated with vehicle (control) or 5 μM AG1478 for 16 hr, then irradiated with indicated dosage. 4 hr after IR, cells were fed with drug-free medium, and incubated for another 20 hr at 37°C, after which they were trypsinized and seeded for clonogenic survival assay. Colony-forming efficiency was determined 14 d later.

As shown in Fig. 3A, LY294002 treatment abolished IR-induced Akt phosphorylation, indicating that this process is dependent upon PI3K, which is consistent with other reports [22]. In addition, treatment with LY294002 significantly increased the radiosensitivity of U87MG cells (Fig. 3B). For example, 47.1% and 93.0% more cells lost their ability to form colonies following treatment with 6 Gy and 9 Gy IR respectively after PI3K was inhibited as opposed to cells where PI3K signaling remained intact. These results indicate that inhibition of PI3K signaling could play an important role in modifying the response of GBMs to IR treatment, consistent with previous observations using U251MG cells [27].

As we had previously shown that EGFR activation was required for IR-induced Akt activation (Fig. 2B), we next tested to whether EGFR signaling modulated radioresistance in U87 cells. To do this, we pre-treated U87MG cells with the EGFR inhibitor AG1478, then treated them with various doses of IR and performed clonogenic survival assays. As depicted in Fig. 3C, inhibition of EGFR had the expected effect of enhancing the radiosensitivity of U87 cells, consistent with its effect on IR-induced Akt activation.

### Genetic inhibition of PI3K signaling enhances the radiosensitivity of U87MG cells

In addition to abolishing PI3K activity, LY294002 has been reported to inhibit other PI3K-like kinases (PIKK), such as mTOR (mammalian target of rapamycin), DNK (DNA-dependent protein kinase), and ATM (ataxia telagiectasia mutated protein) [36]. These kinases play important roles in IR-induced DNA damage repair [37-39], and mTOR regulates the PI3K-Akt signaling pathway at multiple levels [40-43]. As such, it remained possible that the effect of LY294002 on radiosensitivity was independent of its effect on PI3K signaling. Therefore, a genetic approach was used to specifically modulate PI3K-Akt activation and determine the effect on radiosensitivity.

U87MG cells have mutant PTEN genes [34], leading to a high level of Akt phosphorylation. To modulate Akt activation in these cells, genetically modified versions of U87MG cells harboring tetracycline-inducible wild-type or mutant PTEN transgenes were studied.

**Figure 4:**
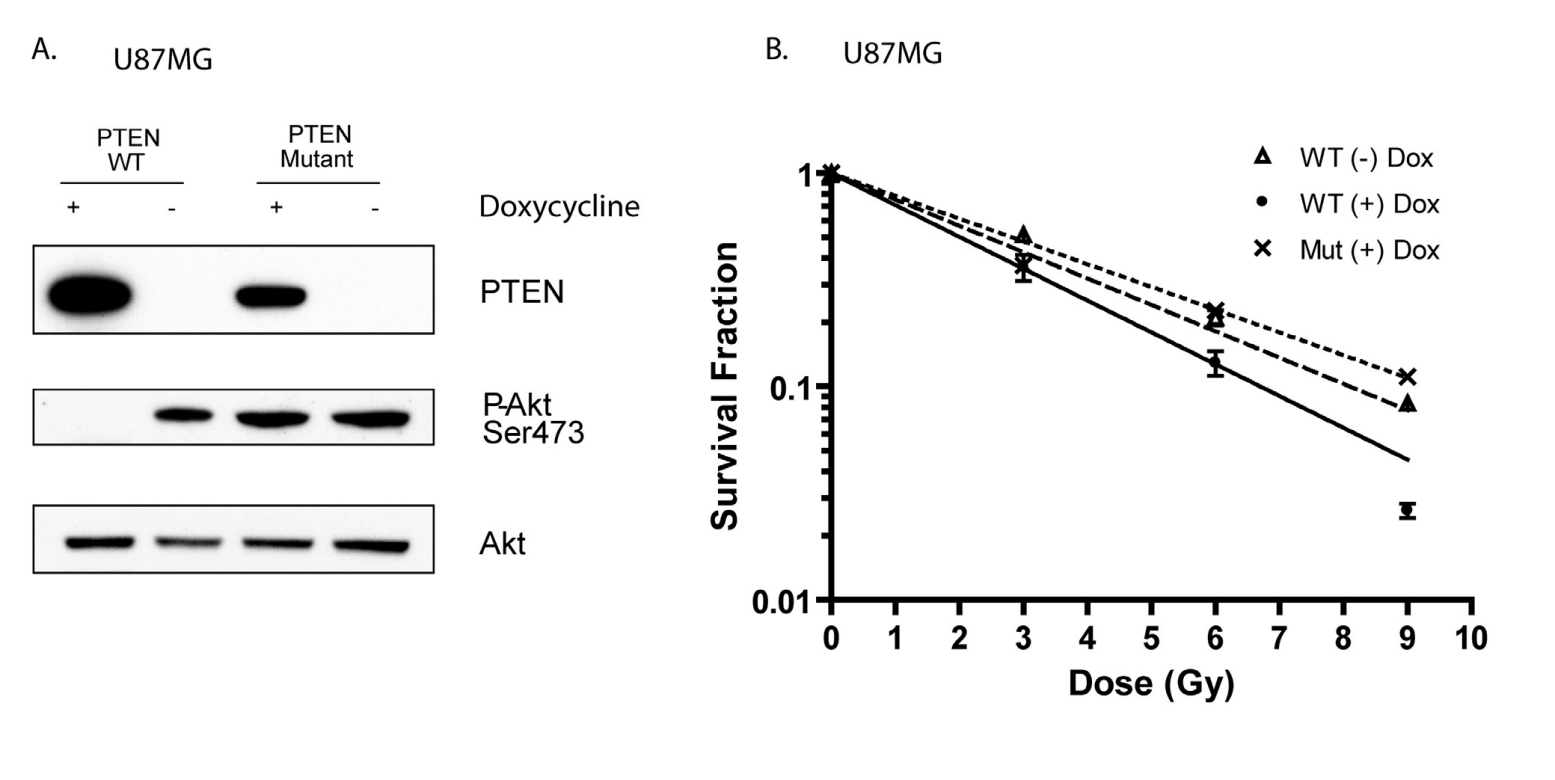
**Expression of PTEN increases the radiosensitivity of U87MG cells**. **A**. Genetically modified U87MG cells were treated with vehicle or 1 μg/mL doxycycline for 24 hr before harvest. Cell lysates were subjected to Western blot analysis with indicated antibody. **B**. Genetically modified U87MG cells were treated with 1 μg/mL doxycycline for 24 hr, and then irradiated with the indicated does. Afterwards, cells were incubated for 4 hr at 37°C, and trypsinized and seeded for clonogenic assay. Colony-forming efficiency was determined 14 d later.

In the absence of doxycycline treatment, both cell lines expressed little PTEN protein and high levels of phospho-Akt (Fig. 4A). Treatment for 24 hr with doxycycline induced robust expression of both wild-type and mutant PTEN, and only the induction of wild-type PTEN led to a significant decrease in Akt phosphorylation (Fig. 4A), confirming that functional PTEN is required for inhibiting Akt activation in GBM.

Next, these U87MG clones were treated with or without doxycycline for 24 hr, followed by radiation treatment. 4 hr after IR, the cells were trypsinized and subjected to clonogenic survival assays. As shown in Fig. 4B, expression of wild-type but not mutant PTEN enhanced the radiosensitivity of U87MG cells. This result is consistent with the results from LY294002 as well as reports from Jiang et al [26], and confirms that IR-induced Akt activation contributes to the radioresistance of U87MG cells.

### Pharmacological inhibition of Akt enhances the radiosensitivity of U87MG cells

Next, we used Akt inhibitors to directly inhibit IR induced Akt activation, and assessed the effect on radiosensitivity. Two different Akt inhibitors, SH-5 and MK-2206 were tested.

**Figure 5:**
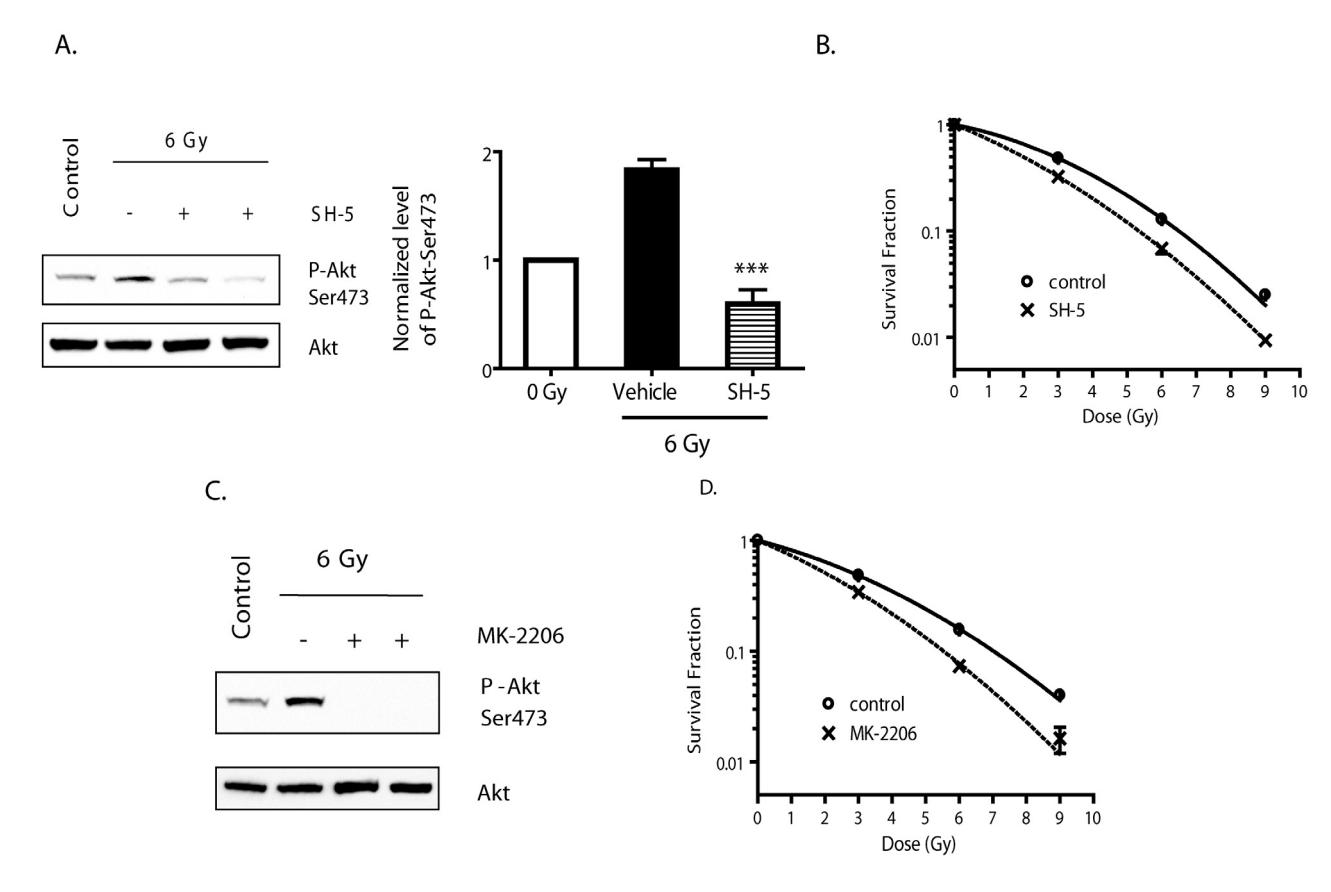
**Akt inhibitors increase the radiosensitivity of U87MG cells**. **A**. U87MG cells were treated with vehicle or 10 μM SH-5 for 16 hrs, and then irradiated with 6 Gy. Total cell lysate was harvested 1 hr after IR and subjected to Western blot analysis with the indicated antibody. Cells without IR treatment were used as a control. The relative ratio of P-Akt-Ser473 to total Akt pooled from two different experiments are shown in the right panel. Results represent mean ± SEM, ***:*p *< 0.001 compared to vehicle (one-way ANOVA) **B**. Cells were treated with vehicle (control) or 10 μM SH-5 for 16 hrs, then irradiated with indicated dosage. 4 hr after IR, cells were fed with drug-free medium, and incubated for another 20 hr at 37°C, after which they were trypsinized and seeded for clonogenic survival assay. Colony-forming efficiency was determined 14 d later. **C**. U87MG cells were treated with vehicle or 1 μM MK-2206 for 1 hr, and then irradiated with 6 Gy. Total cell lysate was harvested 1 hr after IR and subjected to Western blot analysis with the indicated antibody. Cells without IR treatment were used as a control. **D**. Cells were treated with vehicle (control) or 1 μM MK-2206 for 1 hr, then irradiated with indicated dosage. 4 hr after IR, cells were fed with drug-free medium, and incubated for another 20 hr at 37°C, after which they were trypsinized and seeded for clonogenic survival assay. Colony-forming efficiency was determined 14 d later.

SH-5 is a structurally modified phosphatidylinositol ether lipid analogue (PIA) that binds to the PH domain of Akt [44]. SH-5 has been shown to inhibit Akt activation in NSCLC H157 cells with IC_50_~4 μM [44,45]. We found that overnight incubation with 10 μM SH-5 led to a decrease in phospho-Akt in U87MG cells. Therefore, U87MG cells were incubated with 10 μM SH-5 for ~16 hrs, followed by irradiation at 0 - 9 Gy. SH-5 were removed 4 hr after IR, and the cells were further incubated overnight, after which were harvested for clonogenic survival assay as described in the Methods. As shown in Fig. 5A, SH-5 treatment abolished IR-induced Akt phosphorylation without changing the total protein levels of Akt. Consistent with this, treatment with SH-5 increased the radiosensitivity of U87MG cells (Fig. 5-B).

Another tested Akt inhibitor MK-2206 showed similar effect. MK-2206 is a potent allosteric Akt inhibitor with IC_50 _at 8 nm, 2 mM, 65 mM for Akt1, Akt2 and Akt3 respectively [46]. 1 hr treatment of 1 μM MK-2206 abolished Akt phosphorylation in U87MG cells (data not shown). U87MG cells were preincubated with 1 μM MK-2206 for 1 hr, followed by irradiation at 0 - 9 Gy. As shown in Fig 5-C, MK-2206 treatment abolished IR-induced Akt phosphorylation. Moreover, treatment with MK-2206 also increased the radiosensitivity of U87MG cells (Fig. 5-D).

Taken together, these results indicate that Akt is an important downstream effector of PI3K signaling in modifying the response of human GBMs to IR treatment.

## Discussion

Our results demonstrate that irradiation leads to activation of the Akt signaling pathway in a subset of GBM cell lines. IR-induced Akt activation was dependent upon the presence of serum factors, and could be inhibited by the EGFR inhibitor. Inhibiting PI3K, EGFR and Akt activation during irradiation increased the radiosensitivity of U87MG cells.

The U87MG cell line is frequently used as a GBM model, and contains wild-type p53 and mutant PTEN. Our results show that IR induces Akt activation without changing levels of total Akt. However, this effect is substantially less robust in serum-free medium. The fact that radiation-induced Akt activation depends on the presence of serum factors suggests that activation of growth factor receptors is involved in this process.

Overexpression of EGFR is one of the most prominent abnormalities associated with GBMs. Approximately 50% of GBMs contain over-active EGFR, typically through EGFR gene amplification or the expression of an active EGFR mutant. The expression of EGFRvIII, a common constitutively active EGFR mutant, increases radioresistance in immortalized normal human astrocytes [10]. Clinical studies have also shown that EGFR promotes resistance to radiation in many tumor types, including GBMs [10]. Although we did not demonstrate the direct activation of EGFR by IR in this study, this observation has been reported by others. For example, Bowers et al reported that radiation induces EGFR tyrosine phosphorylation in MDA-MB-231 human breast cancer cells minutes after irradiation [35]. Considering that the EGFR inhibitor AG1478 significantly reduced IR-induced Akt activation, it is conceivable that IR induces PI3K-Akt activation through EGFR activation.

Increased Akt activation is associated with radiation resistance in various tumor types. However, most experiments have compared the radiosensitivity of cells with different levels of basal Akt activation [10,47,48]. Since active Akt promotes cell proliferation and inhibits apoptosis, cells with elevated basal Akt activation usually have much higher clone formation efficiency. For example, we found that in medium containing doxycycline, the plating efficiency was much lower in U87MG cells expressing wild-type PTEN as opposed to mutant PTEN genes (data not shown). To account for this difference, our study focused on the effect of IR-induced Akt activation instead of basal Akt activation. Therefore, Akt activation was only inhibited by treatment with a drug, or with an inducible mutant, for a short period of time before and after irradiation, such that Akt activation was not altered during clone formation and clone formation efficiency remained constant. Using U87MG cells we showed that inhibiting IR-induced Akt activation increases radiosensitivity. It is possible that Akt participates in a feedback loop whereby activation of Akt induced by IR increases the radioresistance of GBM cells.

Among the eight GBM cell lines tested for Akt activation, both LN18 and LN229 contain wild-type PTEN, and irradiation induced Akt activation in LN18 cells, but not in LN229 cells. All of the other six GBM cell lines contain mutant PTEN, but the effects of radiation on Akt activation were not consistent. Further experiments are needed to determine if activation of Akt by irradiation is related to the genetic status of PTEN or other factors critical to this signaling pathway.

## Conclusion

In conclusion, our findings indicate that Akt activation may have a critical role in radiosensitivity in a subset of GBM cells. Selective inhibitors that specifically target Akt signaling may have important therapeutic implications when used in combination with radiation in the treatment of GBM patients.

## Competing interests

The authors declare that they have no competing interests.

## Authors' contributions

HL performed experiments, HL, JSK, and TW designed experiments, and HL and TW wrote the manuscript. All authors have reviewed and approved the manuscript.
